# Molecular Research in Chronic Thromboembolic Pulmonary Hypertension

**DOI:** 10.3390/ijms20030784

**Published:** 2019-02-12

**Authors:** Isabelle Opitz, Michaela B Kirschner

**Affiliations:** Department of Thoracic Surgery, University Hospital Zurich, 8091 Zurich, Switzerland; isabelle.schmitt-opitz@usz.ch

**Keywords:** chronic thromboembolic pulmonary hypertension, pathophysiology, genetic alterations, molecular factors, microRNAs, mutations, biomarkers

## Abstract

Chronic Thromboembolic Pulmonary Hypertension (CTEPH) is a debilitating disease, for which the underlying pathophysiological mechanisms have yet to be fully elucidated. Occurrence of a pulmonary embolism (PE) is a major risk factor for the development of CTEPH, with non-resolution of the thrombus being considered the main cause of CTEPH. Polymorphisms in the α-chain of fibrinogen have been linked to resistance to fibrinolysis in CTEPH patients, and could be responsible for development and disease progression. However, it is likely that additional genetic predisposition, as well as genetic and molecular alterations occurring as a consequence of tissue remodeling in the pulmonary arteries following a persistent PE, also play an important role in CTEPH. This review summarises the current knowledge regarding genetic differences between CTEPH patients and controls (with or without pulmonary hypertension). Mutations in BMPR2, differential gene and microRNA expression, and the transcription factor FoxO1 have been suggested to be involved in the processes underlying the development of CTEPH. While these studies provide the first indications regarding important dysregulated pathways in CTEPH (e.g., TGF-β and PI3K signaling), additional in-depth investigations are required to fully understand the complex processes leading to CTEPH.

## 1. Introduction

**Figure 1:**
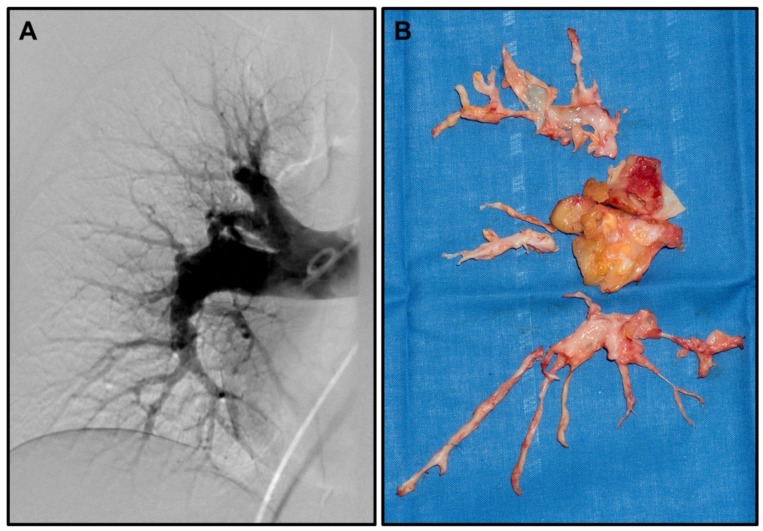
Representative CTEPH angiography and resection specimens: (**A**) Representative pulmonary digital subtraction angiography (Courtesy Prof. T. Frauenfelder) showing pouch-like ending of pulmonary artery segments, as well as stenosis and dilated pulmonary arteries. (**B**) Representative complete resection specimens (right lung) obtained during pulmonary endarterectomy.

Chronic thromboembolic pulmonary hypertension (CTEPH)—as a special form of pulmonary hypertension (PH)—is a rare and disabling disease that can develop as a result of recurring pulmonary embolism (PE) [1,2] (representative pulmonary angiography in Figure 1A). While incomplete or non-resolution of pulmonary thrombi/emboli is the well-accepted main cause of CTEPH in a subset of PE patients [3,4,5], it is unclear what predisposes this subset of patients to develop this rare complication of PH. Furthermore, the exact pathophysiology, including the detailed mechanisms leading to fibrosis and remodelling of the pulmonary arteries, which ultimately lead to CTEPH, remains largely unknown.

The only curative treatment so far is pulmonary endarterectomy (representative photo of resection specimens in Figure 1B), leading to sustained improved quality of life and survival [6,7,8]. However, not all patients are eligible for surgery, leaving many requiring alternative treatments. Here, balloon angioplasty has been suggested, but long-term outcomes for this treatment are still unclear [9]. In addition, alternative medical treatments, such as the endothelin receptor antagonist bosentan, have shown only limited success [10,11]; therefore new treatment avenues are needed for inoperable patients. Furthermore, up to 30% of patients will still have PH even after successful surgery [8], a mechanism not completely understood, but most probably related to secondary distal vasculopathy—another avenue to explore for treatment options and in search of predictive markers for these patients. 

Considering the prominent role of non-resolution of emboli, many studies have investigated the role that altered coagulation, changes in platelet function, and inflammatory reactions may play in the development of CTEPH. Results from these studies have been summarised in detail elsewhere [12,13,14,15], and will therefore only be touched upon briefly here. 

The main focus of this review will be on the underlying genetic/molecular alterations, which have only been started to be investigated in recent years, but which, once elucidated, are likely to help identify biomarkers for early detection and monitoring of patients at risk of developing CTEPH, predicting persistent PH after surgery, as well as potential novel therapeutic avenues.

## 2. Polymorphisms

One of the first described genetic alterations in CTEPH is a polymorphism in the gene encoding for fibrinogen. The study describing this polymorphism was based on the prior identification of several single nucleotide polymorphisms (SNPs) in genes which are relevant for correct fibrinolytic processes, and which had been linked to arterial and venous thrombotic disease [16,17,18,19,20]. In their study, Suntharalingam [21] and colleagues then investigated the frequency of known SNPs in prothrombin, plasminogen activator inhibitor-1, tissue plasminogen activator, Factor XIII, and fibrinogen in a study cohort of 214 CTEPH patients (169 patients with proximal and 45 patients with distal disease) compared to 200 controls. Only one polymorphism in fibrinogen, the Aα Thr312Ala polymorphism, in which the threonine at position 312 of the alpha-chain was being replaced by an alanine, was found to be differentially abundant between the CTEPH and control groups. In addition, the presence of an alanine genotype was associated with an increased risk of CTEPH, although this association did not reach statistical significance in the case of homozygous Aα Thr312Ala polymorphism. The only other polymorphism showing trends for increased odds of CTEPH, as well as an increased frequency in CTEPH, was a Factor V Leiden polymorphism (1691G>A), but these associations were not statistically significant.

The Aα Thr312Ala has also previously been observed in patients with atrial fibrillation, where a higher frequency of post-stroke mortality could be seen in those patients presenting with the fibrinogen polymorphism [20]. The resulting suggestion that the Thr>Ala substitution could be involved in the development of embolic diseases was further confirmed by an in vitro study, which showed that blood clots from subjects with an Ala/Ala phenotype show more α-chain crosslinking and higher clot stiffness [22]. Furthermore, an in vivo study showed that blot clots with the Ala/Ala phenotype are less accessible for fibrinolysis [23], suggesting that this polymorphism could result in a higher aggregation of fibrin and in more tightly packed fibrin-structures, which are less susceptible to fibrinolysis. Indeed, in 2009, Morris and colleagues described that patients with CTEPH frequently show signs of dysfibrinogenemia, abnormal fibrinogen molecules, in the blood [24].

Subsequently, several studies confirmed that increased plasma/serum levels of fibrinogen are detectable in CTEPH (and PH) patients as compared to unaffected controls [25,26], supporting the idea that fibrinogen from patients carrying the Aα Thr312Ala polymorphism (or other polymorphisms affecting the structure of fibrinogen) is indeed more resistant to lysis. Finally, in 2013, Li et al confirmed in a large study including 101 CTEPH, 102 PE (without PH), and 108 healthy controls a higher genotype and allele frequency for the Aα Thr312Ala polymorphism in CTEPH [27]. In addition, the study also showed that the presence of the polymorphism was significantly associated with a higher resistance to fibrinolysis. This resistance was also associated with the specific genotype, with the Thr/Thr genotype and the Thr/Ala genotype showing comparable normal fibrinolysis, while the Ala/Ala genotype showed significantly higher levels of resistance to fibrinolysis [27].

While these data certainly highlight the role of altered fibrinolysis in the development of CTEPH, it is very likely that other genetic factors are contributing and predisposing to the development of CTEPH. Not many studies have so far been undertaken to elucidate these underlying genetic and molecular alterations that could contribute to CTEPH, but some first promising results have been obtained, which are discussed below and summarised in Table 1.

Similar to the results from the study by Suntharalingam et al [21], a study by Ulrich and colleagues [28], which looked into polymorphisms other than those in fibrinogen, namely in bone morphogenetic protein receptor 2 (BMPR2), serotonin transporter (5-HTT) or receptor (5-HTR-2A), and the endothelial nitric oxide synthase (eNOS) genes, did not identify any significant association between the presence of a polymorphism and the presence of CTEPH or the clinical presentation of the patients.

The only other polymorphisms that have been shown to be significantly associated with CTEPH have been described by Xi and colleagues [29]. As part of the mutation analysis of seven PH-related genes in CTEPH patients, this study identified two previously described SNPs (rs3739817 in the endoglin gene and rs55805125 in the MAPK10 gene) that were significantly associated with CTEPH. Further investigations of these SNPs are required to confirm their contribution to CTEPH and to decipher their potential role in the development of the disease.

## 3. Mutations

In PH, it has been shown that not only vasoconstriction and remodelling of the pulmonary arteries caused by, e.g., hypoxia, inflammation, and toxins, are responsible for the development of the disease, but that a genetic predisposition often also seems to play an important role [30]. In the case of hereditary PH, almost 75% of all patients present with a heterozygous germline mutation in the bone morphogenetic protein type II receptor (BMPR2). The same gene also appears to be responsible for idiopathic PH cases, as heterozygous mutations can also be detected in up to 25% of these patients [31,32,33,34]. BMPR2, a serine/threonine kinase, binds bone morphogenetic proteins (BMPs), which are members of the TGF-β superfamily of ligands, and in this way regulates pathways important during embryogenesis and development, but also for the regulation of homeostasis in adult tissue. BMPR2 itself only functions in a heterodimer complex with a type I receptor (BMPR1), in which, upon the binding of BMPs, BMPR2 leads to the phosphorylation and therefore activation of BMPR1. Activated BMPR1 in turn phosphorylates receptor Smads (R-Smads 1/5/9), which then translocate to the nucleus, where they act as transcription factors and regulate the transcription of various target genes [35,36,37,38,39]. In the context of PH and possibly also CTEPH, the involvement of BMPR2 in the proliferation of pulmonary artery smooth muscle cells (PASMCs) and pulmonary artery endothelial cells (PAECs) is of particular interest. Under normal physiological conditions, BMPR2 promotes the survival of PAECs, therefore protecting the pulmonary arteries from damage. Furthermore, by inhibiting the proliferation of PASMCs, BMPR2 prevents the closing of pulmonary arteries [40]. Hence, when BMPR2 is dysfunctional, e.g., through mutations in the BMPR2 gene, uncontrolled proliferation of PASMCs can lead to arterial closure and therefore to PH.

Apart from BMPR2, other members of the TGF-β-signalling pathways have also been shown to be mutated in patients with PH, highlighting that the TGF-β pathway is important for the correct functioning of pulmonary arteries. In particular, the receptor complexes of the TGF-β pathway appear to be affected in patients with PH. For examples, in 2014, Jones and colleagues described the presence of somatic mosaicism in the Activin A Receptor Type 1 (ACVRL1) gene in a family affected by several cases of PH [41]. ACVRL1, also known as activin receptor-like kinase 1 (ALK1), is one of the type I receptors in the TGF-β signalling cascade, which can interact with BMPR2. Due to a described direct interaction with low-density lipoprotein, it has been suggested that ACVRL1 could be involved in early phases of the development of atherosclerosis [42]. Another receptor family member which has been linked to PH is endoglin (ENG). As an auxiliary receptor of the TGF-β complex, ENG participates in the response to ligands such as TGF-β3, Activin A, and BMP2. The expression of ENG during embryogenesis has been linked to the development of the cardiovascular system [43], hence it may play a role in vascular remodelling. Further downstream of the actual receptors, mutation in SMAD9 (also known as SMAD8), one of the downstream effectors regulating gene transcription in the nucleus, has been linked to defective vascular remodelling in a mouse model [44].

While in particular the link between BMPR2 mutations and PH has been well-described, investigations in CTEPH are sparse. The first study investigating BMPR2 mutations in CTEPH was performed by Suntharalingam and colleagues back in 2007 [45], and sequenced 40 idiopathic PH, 25 distal CTEPH, and 25 proximal CTEPH cases for BMPR2 mutations. In 15% of the PH cases, a mutation in BMPR2 was detectable, no mutations were identified in any of the investigated CTEPH cases. This finding was later confirmed by Ulrich et al [28], who investigated 16 CTEPH cases, of which none displayed BMPR2 mutations. However, among these 16 patients, eight showed polymorphisms in the BMPR2 gene, which had previously been described in PH [46]. However, in 2014, a case report was published by Feng and colleagues that described a germline mutation in exon 12 of BMPR2 (c.2296 A>G) in a 29-year old female CTEPH patient [47]. This first report was soon after followed by possibly the most comprehensive sequencing study undertaken so far, conducted by Xi and colleagues in 2016 [29]. Based on the information available for PH, the authors screened 49 Chinese CTEPH patients and 17 control patients with prior PE but without signs of PH, for mutations in seven genes (BMPR2, ACVRL1, ENG, SMAD9, CAV1, KCNK3, and CBLN2). In total, 25 non-synonymous mutations could be detected in the CTEPH patients, and the majority of detected mutations were classified as probably damaging by PolyPhen-2 software. The overall mutation frequency was much higher in CTEPH patients, with 19 of the 49 patients (38.8%) presenting with at least one mutation, as compared to only three of the 17 PE without PH controls (17.6%). Among the investigated genes, the overall mutation rate was the highest in BMPR2, with nine individual mutations being detected. Six of these mutations were located in exon 12, which encodes for the cytoplasmic tail of the receptor, which is essential for signal transmission to the type 1 receptor and then onwards to the SMADs. Somewhat surprisingly, the study also identified a BMPR2 mutation in one of the PE without PH controls. This mutation, located in exon 3, which encodes for the extracellular ligand binding domain, was classified as possibly damaging by PolyPhen-2. While a family history of PH was not known for this patient, it is of course possible that during long-term follow-up, either PH or CTEPH may develop in this individual. Of the six mutations in ACVRL1, five were located in the exons encoding for the kinase domain (one mutation in exon 8, four mutations in exon 10), and one was located in exon 5 encoding for the glycine-serine domain. Both domains are essential for correct signalling activity. Of particular interest is the fact that the mutation detected in exon 10 in four patients is identical to one that has previously been identified in a family with PH in combination with hereditary hemorrhagic telangiectasia [48]. Of the genes directly involved in TGF-β signalling, SMAD9 showed the lowest mutation frequency, with only three individual mutations being detected, with two of those predicted to be probably damaging. 

Mutations in CAV1 were detected in five CTEPH patients; however, only one of those was classified as possibly damaging missense mutation, while the remaining mutations either resulted in variants of unknown significance or were synonymous. Furthermore, one missense mutation in KCNK3 (potassium channel subfamily K, member 3) could be detected in one CTEPH patient. As potassium channels are important players in the regulation of the resting potential of PASMCs, and have been suggested to be involved in pH-dependent hypoxic vasoconstriction [49], it is plausible to speculate that this mutation may indeed be contributing to the development of CTEPH. 

In addition to point mutations, the study also identified nine large size rearrangements, predominantly in the ENG gene, but also one in the BMPR2 gene. Interestingly, the large size rearrangements in ENG were equally common in CTEPH patients and PE without PH controls, while the BMPR2 rearrangements were limited to CTEPH patients. 

Taken together, this study has shown that mutations and genetic alterations previously linked to PH are also detectable in CTEPH patients, and may therefore, in combination with other triggers (e.g., the occurrence of a persistent PE), contribute to the development of CTEPH. However, one has to take into consideration that the two studies identifying BMPR2 mutations in CTEPH patients have screened patients from the Chinese Han population, while the studies investigating BMPR2 mutations in Caucasian CTEPH patients have failed to detect any BMPR2 mutations in these patients [28,45]. This shows that additional, more comprehensive studies in larger patient cohorts are required to fully elucidate the frequency of the described mutations in CTEPH patients, as well as to identify any racial differences in the occurrence of these mutations. In addition, more detailed investigations and a better understanding of the effect that these mutations have on either PAECs or PASMCs is required, to clarify the pathophysiology of CTEPH further. A particular interest here could also be the untranslated regions of the investigated genes. According to a statement by Xi and colleagues [29], additional mutations were also identified in untranslated regions of the investigated genes. While apparently none of these mutations were non-synonymous, future studies should also investigate these untranslated regions, as missense mutations may not cause changes in amino acids, but may still have the potential to alter, for example, the binding capability of microRNAs responsible for post-transcriptional regulation of these genes. 

## 4. Gene Expression

In addition to mutations, altered gene expression in either PAECs or PASMCs could also be a contributor to the development of CTEPH. While altered gene expression has been linked to many other physiological conditions, such as cancer, not many studies have been undertaken in CTEPH thus far. Nevertheless, in 2014, Gu et al [50] performed Affymetrix Gene Chip Arrays on cDNA obtained from PAECs collected from five CTEPH patients and gene expression profiles were compared to those of five controls (donors for lung transplantation). The gene expression analysis was complemented by Gene Ontology (GO) analysis, as well as KEGG pathway analysis. In this small set of patient samples, a total of 1614 genes was found to be differentially expressed between CTEPH and controls (880 genes up and 734 genes down in CTEPH). Neither of the top differentially expressed genes presented in the publication have been linked to CTEPH prior to this study. Nevertheless, taken together, the information obtained from gene expression, GO, and pathways analysis suggests an enrichment in differential expression in cell proliferation, signal transduction, and cytokine-related pathways. The authors particularly highlight the mitogen-activated protein kinases (MAPK) pathway, as well as the phosphoinositide 3-kinase (PI3K) pathway. While investigations in CTEPH are missing, a study by Wei and colleagues [51] has suggested that JNK, a member of the MAPK pathway responsible for response to stress stimuli (e.g., cytokines), is important for the proliferation and migration of PASMCs and could act as a modulator between the MAPK and the PI3K pathways. Both MAPK and PI3K pathways are important players in the regulation of cell proliferation, survival, motility, and cell cycle progression [52,53], and both have been shown to be frequently dysregulated in cancer. Targeting of both MAPK and PI3K pathways is in clinical evaluation for various cancer types [54,55,56,57]; hence, if confirmed to play a role in CTEPH pathophysiology, these pathways represent attractive targets for therapeutic intervention.

While the patient number enrolled in the study by Gu and colleagues is too small to draw a firm conclusion, the obtained data show that gene expression analysis in CTEPH has the potential to identify genes, which, when dysregulated, are contributing to the development of CTEPH. However, additional studies, ideally combined with in vitro functional investigations of identified differentially expressed genes, are required to further unlock the value of gene expression analysis in CTEPH.

## 5. MicroRNAs

MicroRNA are short (~22 nucleotides) non-coding RNAs, which act as important post-transcriptional regulators of gene expression. Through sequence-specific interaction with target sites in the 3’ untranslated region (3’UTR) of messenger RNAs (mRNA), microRNAs regulate the levels of mRNA available for translation, by either inducing mRNA degradation (complete homology with target sequence) or repression of translation (incomplete sequence homology) [58]. Thus far, over 1000 microRNAs have been identified and validated, and these form a complex network of tight control of normal physiological processes, where each microRNA regulates multiple mRNAs and each mRNA is regulated by multiple microRNAs [59,60]. Consequently, dysregulation of microRNA expression has been linked to many disease states, including cardiovascular conditions (reviewed in [61]) and PH [62,63,64].

The first study to link microRNAs to CTEPH was performed by Chen and colleagues in 2010 [65]. The study investigated the association between two of the three known polymorphisms in fibrinogen A (FGA), the -58A-Insertion in the 5’UTR and the 28bp insertion/deletion (indel) region in the 3’UTR of the FGA gene, and CTEPH. Since microRNAs target the 3’UTR of mRNA, additional target prediction analysis was performed, which revealed microRNA miR-759 as a candidate for targeting the indel region in the 3’UTR. Indeed, further analyses then suggested this microRNA to be specifically expressed in liver tissue, and therefore at the location of fibrinogen production. Furthermore, targeting of FGA indel by miR-759 was confirmed in in vitro experiments, and based on their data, the authors concluded that the presence of miR-759 results in degradation of the long isoform of FGA mRNA. 

In 2013, a small microarray profiling study by Wang and colleagues [66] investigated the expression of microRNAs in PASMCs obtained from surgical specimens. The authors identified 12 microRNAs as upregulated and six microRNAs as downregulated in PASMCs from CTEPH patients (N=5) as compared to PASMCs from control individuals (N=3). In silico analyses consisting of target prediction and investigation of the regulated mRNA network, ranked microRNAs let-7c, miR-27, and let-7d the highest, suggesting these as key microRNAs in CTEPH. Using RT-qPCR as an alternative technique, only the downregulation of let-7d could be validated, while levels of let-7c were the same in PASMCs from CTPEH and control patients, and miR-27 was not validated by RT-qPCR. Upon transfection with a let-7d mimic, PASMCs showed a reduced proliferation, as well as an upregulation in p21 protein levels. While no further investigations were performed, based on these data, the authors speculated that inhibition of p21 as a result of low let-7d levels leads to the promotion of PASMC proliferation. However, this information, as well as the fact that the two predicted let-7d target genes TGFBR1 and p21 are present at lower levels in PASMCs from CTPEH patients with low let-7d levels, is somewhat contradictory as microRNAs lead to the repression of translation or degradation of their target genes, meaning in cases of low let-7d, p21 and TGBR1 should be upregulated. Hence, further in depth-investigations of the let-7 family are needed before their role in CTEPH pathophysiology can be confirmed. 

The remaining studies investigating microRNAs in CTEPH have all started from peripheral blood samples, with only one of them performing additional functional analyses of the identified microRNAs. 

Miao and colleagues published two studies in 2017, which used the same patient samples (five CTEPH vs five controls) for microarray-based profiling [67,68]. The first study profiled the circular RNA (circRNA) content of whole blood, followed by the prediction of circRNA-microRNA interactions [67]. These interactions are relevant since circRNAs have been shown to act as “microRNA sponges” and are therefore thought to be involved in the regulation of microRNA target genes. Of the 351 differentially expressed circRNAs, 23 were associated with >100 microRNAs each, and ultimately, miRs-939-5p, -942-5p, -940, and -92a-2-5p appeared to be the microRNAs associated with several of the identified circRNAs. Based on GO and KEGG pathway enrichment analyses of both circRNAs and target microRNAs, the authors ultimately suggested the circ-0002062-miR-942-5p-CDK6 pathways involved in cancer and the circ-0022342-miR-940-CRKL-Erb signalling pathway axes as important for pathways in CTEPH. However, these hypotheses are solely based on in silico analyses combined with literature searches, with actual confirmation of the importance of these pathways completely missing. In their second study, Miao and colleagues then performed microRNA profiling in whole blood RNA from the same patients, which identified 24 up- and 22 downregulated microRNAs in CTEPH [68]. Interestingly, none of these microRNAs overlap with those identified in the circRNA study. The authors then selected the microRNA with the highest number of predicted target genes (miR-3148) for validation using RT-qPCR in the same samples, but again, no further investigations to confirm the role of this microRNA were performed.

The most comprehensive microRNA study thus far was performed by Guo and colleagues in 2014 [69]. Initial profiling of plasma from 10 CTEPH patients and 10 healthy controls identified 15 up- and 20 downregulated microRNAs, and a combination of 17 of those provided diagnostic sensitivity and specificity of >90%. Through target prediction and GO analysis, the authors found the most important dysregulated pathways to be associated with cell proliferation. Furthermore, immunity-related and cell function pathways, such as cell adhesion and migration, were upregulated, while cardiovascular-related pathways and calcium signaling were generally downregulated. Following a literature review, three microRNA candidates, the upregulated oncogenic miR-602 and the downregulated tumor-suppressive miR-22 and let-7b, were validated in plasma samples from an additional 40 patients and controls, respectively. While the upregulation of miR-602 could not be confirmed, both miR-22 and let-7b levels were significantly lower in plasma from CTEPH patients, and both showed a moderate diagnostic accuracy, with AUCs of 0.75 (miR-22) and 0.77 (let-7b), respectively. For let-7b, the authors also described the expression differences between patients with and without acute vascular reaction, as well as the positive correlation with plasminogen activator inhibitor 1 (PAI-1) and D-Dimer, and a negative correlation with the cardiac index of the patients. As in silico target prediction then identified ET-1 and TGFBR1 as potential target genes of let-7b, this microRNA was investigated in more detail, and both genes could be confirmed as direct let-7b targets. For ET-1, a key vasoconstrictor implicated in CTEPH pathophysiology, a negative correlation with let-7b levels could be shown, meaning that ET-1 was significantly increased in patients with low let-7b levels. In vitro investigations in both PAECs and PASMCs indicated that let-7b is a regulator of the expression of TGFBR1 and ET-1, and that in particular, through TGFBR1, let-7b regulates the migration of both PAECs and PASMCs. 

Taken together, these data suggest that in particular, let-7b through its targets ET-1 and TGFBR1, which have previously been implicated in CTEPH pathophysiology, but also other microRNAs, might be involved in the regulation of important pathways affecting the cells in the pulmonary vasculature in a way that ultimately leads to the development of CTEPH. Further studies in this area, in particular a detailed functional investigation of the identified microRNAs, are urgently needed in order to further elucidate the role that microRNAs play in the pathophysiology of CTEPH.

However, while microRNAs are certainly likely major contributors to the development of CTEPH and hold strong potential as useful biomarkers and potential therapeutic targets, research in the field of microRNAs is somewhat still in its infancy and many challenges remain to be tackled before microRNAs will ultimately find their way into routine clinical use (reviewed in [70,71,72]). For example, is it necessary to understand the complexity of the microRNA-mRNA network in order to understand the consequences that therapeutic altering of microRNA expression may have on the whole organism [70,72]. Furthermore, while having shown promise as biomarkers, a robust method for their detection, in particular in the circulation, still needs to be developed [71].

## 6. DNA Methylation

Quite recently, the first study investigating DNA methylation patterns in PASMCs was published by the group of Miao and colleagues [73], who have also investigated microRNAs and circRNAs in CTEPH plasma samples. Methylation array profiles of five primary PASMC lines generated from endarterectomy specimens from five CTEPH patients were compared to those of three commercially available normal PASMC lines. GO analysis revealed that the 775 hypermethylated genes were enriched in function terms associated with morphogenesis and cytoskeleton organisation, and the 446 hypomethylated genes were enriched in actin-related processes, cell-matrix adhesion, and apoptosis. Interestingly, similar to the data from the microRNA and circRNA studies, KEGG pathway analyses showed high enrichment scores for cancer-related pathways, but as expected, also for regulation of the actin cytoskeleton. Validation of six selected genes showed that for 50% of these genes, the methylation status correlated with a significant down- or upregulation of the respective mRNA in an additional five control and 11 CTEPH PASMC cell lines. 

## 7. Transcription Factor FoxO1

Similar to DNA methylation, the role of aberrant transcription factor expression has thus far only been studied by one group [74,75]. In 2016, Deng and colleagues used a rat model in which CTEPH was induced by repeated injection of blood clots into the jugular vein, to investigate the expression of the transmembrane glycoprotein tissue factor (TF) and the transcription factor forkhead box transcription factor O-1 (FoxO1) [74]. TF is important in the initiation of the extrinsic coagulation pathways, while FoxO1 is responsible for the regulation of genes involved in inflammation, cell proliferation, and apoptosis. Reduced FoxO1, previously suggested to contribute to the development of PH in a rat model, was also observed in rats developing CTEPH, while TF levels in the plasma increased with CTEPH development. In addition to the negative correlation with TF, FoxO1 levels also showed a trend towards a negative correlation with mean pulmonary artery pressure (mPAP), but any underlying mechanisms were not investigated in this study. However, in a second study published later in 2016 [75], the same group showed that together with the decrease of FoxO1 expression in the four-week period of CTEPH development, anti-apoptotic Bcl-2 also decreased, while pro-apoptotic Bad levels were increased in the PAECs. In addition, Bcl-2 levels negatively correlated with mPAP, and Bad levels showed a positive correlation with mPAP. These data, while not proving a direct link between FoxO1 and Bcl-2 and Bad, provide a hint that the loss of FoxO1 is accompanied by an induction of apoptosis in PAECs. However, more in-depth analyses are again required to confirm this hypothesis.

## 8. Summary and Outlook

Overall, studies investigating the underlying molecular and genetic alterations responsible or contributing to the development of CTEPH are sparse, and validation studies are usually not available. In addition, when specific alterations, in particular mutations in the BMPR2 gene, have been investigated by several authors, the presented data are contradictory. It is of importance to note that the majority of studies discussed here have been performed in Chinese Han populations, while only a few sequencing and profiling studies have been embarked on in Caucasian populations. This imbalance should be tackled in the future as the differences in study populations could possibly explain the discrepancies, e.g., for BMPR2 mutations. Equally important is the inclusion of comprehensive clinical data of the investigated samples, especially when aiming to identify novel biomarkers. The majority of the studies undertaken thus far have solely reported on the expression of any identified candidate, but correlations between the occurrence or abundance level of, e.g., a microRNA, with the clinical presentation of the included patients (e.g. mean pulmonary arterial pressure, Jamieson classification, or performance in six-minute walking tests) are completely lacking, therefore not allowing any conclusion regarding the relationship between the proposed biomarkers and the severity of the disease. However, this information is crucial, especially when we are considering using biomarkers for the monitoring of disease progression and response to therapy. Furthermore, in particular, the studies performing large scale profiling, e.g., microRNA arrays, gene expression arrays, and methylation profiling, have only included very small numbers of patients and controls, and mostly lack the inclusion of independent larger validation cohorts. While CTEPH is a rare disease, larger sample sets and independent validation are needed to confirm the findings obtained from profiling a handful of patients. To achieve this, international collaboration between research groups and specialised centres for CTEPH should be promoted. However, in order to really understand the pathophysiology of CTEPH, it will not suffice to perform large-scale profiling/sequencing studies to identify biomarkers for diagnosis, prognosis, or response to a known therapy. It is also crucial to include functional investigations of the identified CTEPH-related molecular alterations, as this is the only way we can really elucidate and understand the mechanisms underlying the development of the disease, which will ultimately not only provide us with a better understanding of the pathophysiology of CTEPH, but is also likely to enable us to identify novel therapeutic strategies, in particular, targeted therapies. 

Furthermore, it should not be forgotten that any study, preclinical or clinical, needs to be thoroughly designed to allow firm conclusions to be drawn and to ensure reproducibility of the obtained data. It is of the utmost importance that preclinical animal studies use appropriate sample size calculations, predefinition of measured outcomes, standardised methods for data collection and analysis, clearly defined study populations, and transparent data reporting, to allow the translation of obtained results into clinical care. Similarly, studies with patient material and clinical studies have to be thoroughly designed. While in-depth discussions of the specific items to be considered are beyond the scope of this review, extensive work in PH provides detailed information, which can be easily used and adapted for CTEPH research [76,77].

Nevertheless, while more comprehensive, rigorously designed large-scale studies are certainly required, the available literature shows us that there is a strong rationale for comprehensive genetic studies, ideally not only on blood samples, but also including PAECs and PASMCs obtained from CTEPH patients, as, like in other diseases, these studies have the potential to unveil novel insights into dysregulated pathways involved in the development of CTEPH. For example, several of the studies, which also employed GO and KEGG pathways analysis, suggested common pathways to be affected in CTEPH. Among these are the TGF-β, PI3K, STAT, and MAPK signalling pathways, which are all involved in the regulation of cell growth, proliferation, and survival, but also cell migration and cell adhesion; hence processes which are likely to be dysfunctional in a disease which is associated with remodelling of the pulmonary arteries. In addition, besides GO terms related to the organisation and integrity of the actin cytoskeleton, cancer-related pathways have frequently been identified as being enriched in CTEPH patients’ altered gene or microRNA expression. Again, these pathways represent very interesting candidates for further investigations, as the changes occurring during the development in CTEPH, which involve remodelling of the pulmonary arteries and most likely changes in the behaviour of both PAECs and PASMCs, are similar to those observed in cancer, in particular during the processes leading to metastases at distant sites. 

Taken together, thus far, only limited information regarding the genetic and molecular features of CTEPH, beyond abnormal fibrinolytic and coagulation processes, is available. However, a better understanding of underlying genetic abnormalities, in particular in PAECS and PASMCs, holds the potential to not only elucidate further the pathophysiology of CTEPH, but to also identify novel biomarkers and importantly novel therapeutic avenues for the treatment of this debilitating disease. Hence, fostering collaborations between research centres to allow screening and profiling, as well as validation of obtained data, in larger patient cohorts is urgently needed to enable the CTEPH community to obtain a better understanding of the pathophysiology of CTEPH.

**Table 1:** Overview of genetic alterations proposed to be associated with CTEPH.

Genetic Alteration	Association with CTEPH	Possible Effect of Alteration	Ref.
**Polymorphisms**			
Aα Thr312Ala	Frequency ↑	Resistance to fibrinolysis	[21,24,25,26,27]
rs3739817 (ENG)	Frequency ↑	Unknown	[29]
rs55805125 (MAPK10)	Frequency ↑	MAPK signalling	[29]
**Mutations**			
BMPR2	Frequency ↑	Disrupted TGF-β signalling, induction of PASMC proliferation	[29]
ACVRL1	Frequency ↑	Disrupted TGF-β signalling	[29]
SMAD9	Frequency ↑	Disrupted TGF-β signalling	[29]
CAV1	Frequency ↑	Disrupted TGF-β and nitric oxide signalling	[29]
KCNK3	Frequency ↑	Effect on resting potential of PASMCs	[29]
**Gene Expression**			
Various genes	↑ and ↓	Enrichment of dysregulation in cell proliferation, signal transduction, cytokine-related and cancer-related pathways	[50]
**microRNAs**			
miR-759	Unknown	Degradation of fibrinogen	[65]
Let-7d	↓	Inhibition of PASMC proliferation	[66]
miR-942-5p– circ0002062 axis	↓ in plasma	Dysregulation of CDK6 signalling	[67]
miR-940– circ0022342 axis	↓ in plasma	Dysregulation of Erb signalling	[67]
Let-7b	↓ in plasma	Regulation of TGBFR1 &ET-1 expression + PAEC and PASMC migration	[69]
miR-22	↓ in plasma	Unknown	[69]
**DNA Methylation**			
PIC3CA	↓	Dysregulation of cancer -related pathways	[73]
HFA	↓	Dysregulation and actin cytoskeleton regulation of cancer-related pathways	[73]
HIC1	↑	Dysregulation and actin cytoskeleton regulation of cancer-related pathways	[73]
**Transcription Factors**			
FoxO1	↓	Modulation of apoptosis in PAECs	[75]
